# Robustness of individual and marginal model-based estimates: A sensitivity analysis of flexible parametric models

**DOI:** 10.1016/j.canep.2018.10.017

**Published:** 2019-02

**Authors:** Elisavet Syriopoulou, Sarwar I. Mozumder, Mark J. Rutherford, Paul C. Lambert

**Affiliations:** a Biostatistics Research Group, Department of Health Sciences, University of Leicester, University Road, LE1 7RH, Leicester, UK; b Department of Medical Epidemiology and Biostatistics, Karolinska Institutet, SE-171 77, Stockholm, Sweden

**Keywords:** Flexible parametric survival model, Restricted cubic splines, Relative survival, Cancer, Interactive graphs

## Abstract

**Background:**

Flexible parametric survival models (FPMs) are commonly used in epidemiology. These are preferred as a wide range of hazard shapes can be captured using splines to model the log-cumulative hazard function and can include time-dependent effects for more flexibility. An important issue is the number of knots used for splines. The reliability of estimates are assessed using English data for 10 cancer types and the use of online interactive graphs to enable a more comprehensive sensitivity analysis at the control of the user is demonstrated.

**Methods:**

Sixty FPMs were fitted to each cancer type with varying degrees of freedom to model the baseline excess hazard and the main and time-dependent effect of age. For each model, we obtained age-specific, age-group and internally age-standardised relative survival estimates. The Akaike Information Criterion and Bayesian Information Criterion were also calculated and comparative estimates were obtained using the Ederer II and Pohar Perme methods. Web-based interactive graphs were developed to present results.

**Results:**

Age-standardised estimates were very insensitive to the exact number of knots for the splines. Age-group survival is also stable with negligible differences between models. Age-specific estimates are less stable especially for the youngest and oldest patients, of whom there are very few, but for most scenarios perform well.

**Conclusion:**

Although estimates do not depend heavily on the number of knots, too few knots should be avoided, as they can result in a poor fit. Interactive graphs engage researchers in assessing model sensitivity to a wide range of scenarios and their use is highly encouraged.

## Highlights

• Estimates obtained from a flexible parametric model are not oversensitive to the number of knots used to create the splines.
• Non-proportional hazards can easily be incorporated in the model and the estimates remain non-sensitive.
• Flexible parametric models have advantages for obtaining useful predictions compared to other models, such as the Cox model.
• Online interactive graphs are a powerful tool that enable users to improve understanding of findings.

## 1 Introduction

Flexible parametric survival models (FPMs), which were first introduced by Royston and Parmar, have been used in a range of settings [1,2]. The models have been used to estimate survival in epidemiological studies with applications involving international comparisons [3] and they have also been adapted to clinical trials settings [4]. The methodology has been extended to the relative survival framework in population-based data [5]. It has also been used to assess statistical cure [6] and to estimate the loss in life expectancy due to a cancer diagnosis [7].

Population-based studies that include all patients in a geographically-defined population provide a measure of the effectiveness of the healthcare system in diagnosing and treating the cancers that arise. A commonly reported measure of cancer survival is relative survival, which compares the all-cause survival for a group of cancer patients to the expected survival of a comparable group in the general population that is free of the cancer of interest [8].

An increasing number of population-based studies perform analysis by using FPMs rather than traditional methods [[9], [10], [11]]. The model is fitted on the log cumulative excess hazard scale and directly models the effect of time by using splines. Splines are flexible mathematical functions defined by piecewise polynomials, which under constraints, form a smooth function. The points at which the polynomials join are called knots. The number of knots, or degrees of freedom (df) that is equal to the number of knots minus 1, specified to create the splines determines the number of parameters to model the hazard function [12].

A small debate exists on the number of knots used for the splines. Sensitivity analyses are often conducted to ensure that the df does not influence the estimates. A simulation study, showed that the estimated relative effects are insensitive to the correct specification of the baseline hazard and that, provided enough knots are selected, complex hazard functions can be captured [13]. They also showed that absolute effects are well captured. Another simulation study showed that time-dependent effects can also be captured accurately [14].

We assess how reliable estimates from FPMs are by choosing several df to model the log cumulative baseline excess hazard and the main and time-dependent effect of age using English cancer data. We also developed web-based interactive graphs, where users can compare the estimates from different models.

## 2 Methods

### 2.1 Data resources

Data consist of National Cancer Registry Data, provided by Public Health England, on all individuals in England diagnosed with one of the cancers of interest between the start of 2007 and the end of 2013. We included cancer types with both high and low survival after diagnosis and varying characteristics such as age at diagnosis. The 10 cancer types considered are bladder, lung, colon, rectum, stomach, melanoma, prostate, breast, ovarian cancer and Hodgkin lymphoma. Individuals were identified using International Classification of Diseases 10 (Table 1). For patients with multiple tumours only the first tumour for each type of cancer is considered.

**Table 1:** Number and mean age of cancer patients diagnosed between 2007–2013 in England, per cancer type.

Cancer Type^a^	Gender	N	Age(mean)
Bladder	Males	44,032	73.64
	Females	16,641	75.64
Lung	Males	131,252	71.85
	Females	105,465	71.94
Colon	Males	76,937	71.14
	Females	69,989	72.68
Rectum	Males	50,068	69.09
	Females	30,322	70.42
Stomach	Males	26,996	72.01
	Females	14,318	73.99
Melanoma	Males	35,099	63.06
	Females	37,398	59.33
Hodgkins	Males	5,548	47.37
	Females	4,288	46.9
Prostate	Males	249,184	71.04
	Females	–	–
Breast	Males	–	–
	Females	273,988	62.85
Ovarian	Males	–	–
	Females	39,491	63.89

a International Classification of Diseases 10: bladder cancer (C67), lung cancer (C34), colon cancer (C18), rectum cancer (C19, C20), stomach cancer (C16),), melanoma (C43), Hodgkins lymphoma(C81), prostate cancer (C61), breast cancer (C50), ovarian cancer(C56).

### 2.2 Flexible parametric survival models

In this application, we only consider FPMs that are fitted on the log-cumulative hazard scale rather than the log hazard scale. An advantage of this way of modelling is that the corresponding function is more stable and the process of capturing the shape of the function is easier. FPMs explicitly estimate the log cumulative hazard by using restricted cubic splines for ln(t) [1,2,5,15]. See Appendix A for details on splines. A proportional hazards FPM with knots $k_{0}$ for the log baseline cumulative hazard is

$$\text{ln}\left[H\left(t,x\right)\right]=\;s\left(\text{ln}\left(t\right)|\gamma ,k_{0}\right)\;+x\beta$$

where t is time, $s\left(\text{ln}\left(t\right)|\gamma ,k_{0}\right)$ is a restricted cubic spline function of log time with γ values for the parameters, x are the covariates and β the corresponding coefficients. FPM is an extension of the Weibull model and if only 1 df is used for the baseline hazard function, then fitting a FPM is equivalent to fitting a Weibull model.

This is a proportional hazards model and the interpretation of the covariates is the same as for models on the log hazard scale e.g. a cox model. Non-proportional hazards (time-dependent effects) are easily incorporated in the model by including interactions between covariates and spline functions for log time

$$\text{ln}\left[H\left(t,x\right)\right]=\;s\left(\text{ln}\left(t\right)|\gamma ,k_{0}\right)\;+x\beta +\sum_{j=1}^{D}s\left(\text{ln}\left(t\right)|\delta_{j},k_{j}\right)x_{j}$$

where D is the number of time-dependent effects and $s\left(\text{ln}\left(t\right)|\delta_{j},k_{j}\right)$ is the spline function for the j th time-dependent effect with $\delta_{j}$ values for the parameters.

Modelling time-dependent effects usually requires fewer knots than the baseline effects because we actually model departures from the baseline hazard.

FPMs have been extended to estimate excess mortality and relative survival which are commonly reported measures in cancer epidemiology.

### 2.3 Excess mortality and relative survival

Excess mortality is equal with the difference between the observed (all-cause) mortality in a population of cancer patients and the expected mortality in a comparable group.

Relative survival is the survival analogue of excess mortality and is given as the all-cause survival among the cancer patients divided by the expected survival in a comparable group in the general population with similar characteristics, who are assumed to be free of the cancer of interest [8,16]. The expected survival is considered to be known and is obtained from available life tables.

Relative survival aims to estimate survival in a hypothetical scenario where the cancer of interest is the only possible cause of death and with some assumptions is equivalent to what is known in the statistical literature as net survival, a useful measure for comparing survival between populations, such as countries, or for studying temporal trends [[17], [18], [19]].

A major advantage of relative survival is that it circumvents problems caused by the inaccuracy or non-availability of death certificates as it does not rely on the cause of death information [16].

### 2.4 Sensitivity analysis

We assessed the reliability of relative survival estimates by using data for a range of cancer types. Age was included in the models as a continuous variable but non-linearity was allowed by using splines. Models on population-based studies usually have non-proportional hazards. For example, the effect of age is bigger in the beginning of the follow-up right. Thus, the time-dependent effect of age was included in the models. We chose varying df to model the baseline excess hazard (i.e. 3,4,5,6,7 df) and the main (i.e. 3,4,5 df) and time-dependent effect of age (i.e. 2,3,4,5 df) resulting in 60 FPMs for each cancer type. The knots for the baseline excess hazard and the time-dependent effect of age were placed at equally distributed quantiles of the log of the event times. Additional boundary knots were also placed at the minimum and maximum of the distribution of the log of the event times. Similarly, the knots for the main effect of age were placed in equally distributed quantiles of the age distribution. The model estimates are usually not sensitive to the location of the knots [20]. Expected mortality rates were obtained from population mortality files stratified by sex, age and calendar year [21]

Hodgkin lymphoma affects a smaller portion of the population and is particularly common at younger ages. The small number of events and the different profile of the youngest and the oldest patients caused convergence issues for some models. To enable the models to fit and compare different scenarios, even though we allowed time-dependent effects for the effect of age, these were limited to the linear term of the splines.

For each model we obtained both 1-year and 5-year, age-specific, age-group and internally age-standardised relative survival estimates that are common measures for population-based studies. The age-specific estimates are given for ages 55, 65, 75 and 85. The groups used for the age-group estimates were 18–44, 45–54, 55–64, 65–74 and 75+. For prostate cancer were however obtained for groups 18–54, 55–64, 65–74, 75–84 and 85+, as prostate cancer is frequent in elderly men. Internally age-standardised estimates were estimated as the weighted average of relative survival in each age group, based on the age distribution within our study population. External age standardisation is also possible using weights from standard cancer populations [22].

With continuous data is common to have less stable results in the extremes due to the small number of observations. An issue with relative survival might be that some patients have better survival than expected and this may occur by chance when there are few numbers, such as in the young or elderly. This might lead to negative excess mortality and further issues with the models’ convergence. To make estimates on the extremes more stable and help with convergence problems, we forced patients who were younger than the age corresponding to the 2nd percentile of the age distribution of each cancer type to have the same relative survival as patients of this cut-off age. The same was applied for patients who were older than the age corresponding to the 98th percentile of the age distribution. Thus, 96% of the age distribution was modelled continuously.

The Akaike Information Criterion (AIC) and Bayesian Information Criterion (BIC) were calculated for each model [23,24]. Furthermore, we obtained estimates using the Ederer II and Pohar Perme non-parametric methods that require no modelling assumptions, for comparison [8,17,25]. The model with 5, 3 and 3 df for the baseline excess hazard, the main and the time-dependent effect of age respectively is used as the reference model. This is not consider to be the correct model and it was selected, based on what is usually used in FPMs, to make the illustration and comparison to the AIC and BIC selection criteria easier.

A period analysis with a 3-year period window between 2011 and 2013 was conducted for all the models and therefore the analysis included only the person time that is included in the window. Period analysis has been shown to provide good predictions for the prognosis of recently diagnosed patients [26,27].

All statistical analyses were conducted using Stata 14 [28]. See Appendix C for Stata code.

### 2.5 Interactive graphs

We also developed web-based interactive graphs that help users understand the effect of different df on the estimates by comparing the estimations derived by different models. Survival and hazard functions are given over years since diagnosis and the users can choose the models they are interested in. Both age-standardised and age-specific estimates are provided. A major advantage of interactive graphs is being able to control what information is displayed. Further exploration of findings is enabled and therefore users develops a better understanding of the results.

## 3 Results

Data includes a population of more than 1.2 million cancer patients. Patients with Hodgkin lymphoma are the youngest with the average age to be approximately 47 years (Table 1). The oldest are bladder cancer patients, at the mean age of 76 and 74 years for females and males respectively.

**Fig. 1:**
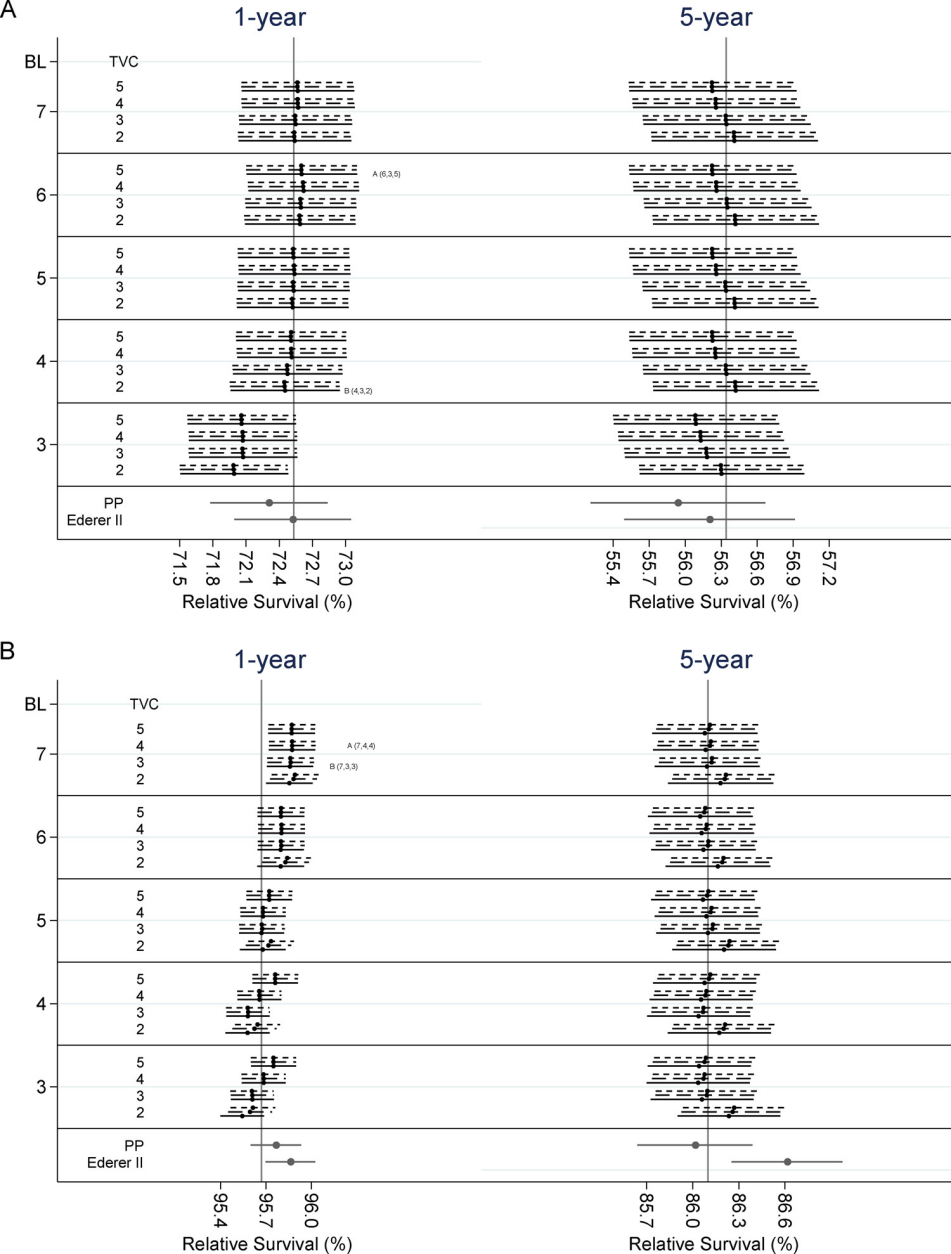
Age standardised estimates for 1-year and 5-year relative survival for A) female patients with colon cancer and B) males with prostate cancer. The dots represent the point estimates and the lines either side the 95% confidence intervals. The vertical line, in each plot, represent the estimate obtained by the reference model. Solid, dash and dotted horizontal lines represent 3, 4 and 5 degrees of freedom, respectively, for the main effect of age. (BL: degrees of freedom for the baseline excess hazard, TVC: degrees of freedom for the time-dependent effect of age, A: model chosen by AIC, B: model chosen by BIC, PP: Pohar Perme estimate).

Table 2A, Table 2B show the differences in the 1-year and 5-year relative survival estimates between the reference model and the model selected by the AIC or BIC criterion. For the age-standardised estimates, absolute differences remain lower than 0.5 percentage point. In specific, absolute differences between the reference model and the model chosen by the AIC criterion, for females, have a median value of 0.083 and a mean of 0.091 percentage points. Similarly, differences with the BIC model have a median and mean equal to 0.80 and 0.110 respectively and differences between the reference model and the Pohar Perme estimates have a median value of 0.182 and mean of 0.213. The age-standardised estimates for colon cancer female patients and prostate cancer patients can also be seen graphically in Fig. 1. The minor differences observed show that the standardised estimates do not depend heavily on the choice of df. The big size of the datasets results in narrow 95% confidence intervals. Detailed plots of standardised relative survival from 18 out of the 60 scenarios can be found in the interactive graphs.

**Table 2A:** Differences between the estimates of survival of the reference model with the one with the minimum AIC and BIC respectively, for females as a whole population (standardised), females in age-groups or females aged 55, 65, 75 and 85 by type of cancer.

Cancer Type	Time (years)	Standardised			Group 1^a^		Group 2^a^		Group 3^a^		Group 4^a^		Group 5^a^		55		65		75		85	
		AIC	BIC	PP	AIC	BIC	AIC	BIC	AIC	BIC	AIC	BIC	AIC	BIC	AIC	BIC	AIC	BIC	AIC	BIC	AIC	BIC
Bladder	1	0.04	0.37	0.39	0.05	0.64	0.05	0.49	0.04	0.23	0.04	0.16	0.04	0.46	0.04	0.34	0.04	0.17	0.03	0.19	0.03	0.50
	5	−0.01	−0.24	0.54	0.00	−0.61	0.00	−0.43	0.00	−0.10	0.00	0.05	−0.02	−0.35	0.00	−0.25	0.00	0.00	0.00	0.05	−0.02	−0.42
Lung	1	−0.24	−0.26	−0.05	−0.47	−0.47	−0.38	−0.38	−0.19	−0.19	−0.15	−0.16	−0.30	−0.33	−0.28	−0.28	−0.14	−0.14	−0.20	−0.22	−0.35	−0.38
	5	0.06	0.07	0.22	0.10	0.09	0.05	0.04	−0.04	−0.05	−0.02	−0.02	0.16	0.18	−0.01	−0.01	−0.06	−0.06	0.05	0.06	0.22	0.24
Colon	1	−0.07	0.08	0.22	−0.23	0.24	−0.38	0.12	−0.46	0.01	−0.30	0.00	0.21	0.12	−0.45	0.06	−0.44	−0.01	−0.07	0.05	0.34	0.16
	5	0.11	−0.08	0.40	0.08	−0.05	0.07	−0.03	0.05	−0.03	0.03	−0.06	0.18	−0.11	0.06	−0.03	0.04	−0.04	0.02	−0.09	0.23	−0.13
Rectum	1	0.03	−0.08	0.13	0.20	0.14	0.09	0.04	0.00	−0.04	−0.04	−0.12	0.07	−0.12	0.04	−0.01	−0.03	−0.08	−0.05	−0.15	0.09	−0.12
	5	−0.09	−0.14	0.09	0.01	−0.07	−0.04	−0.11	−0.02	−0.08	0.11	0.07	−0.27	−0.30	−0.05	−0.11	0.03	−0.02	0.18	0.14	−0.45	−0.48
Breast	1	−0.16	−0.17	−0.08	−0.08	−0.06	−0.05	−0.06	−0.02	−0.07	−0.30	−0.13	−0.33	−0.44	0.05	−0.06	−0.23	−0.09	−0.17	−0.20	−0.18	−0.50
	5	0.03	0.05	0.22	0.01	0.03	−0.10	0.03	0.45	0.02	−0.75	0.04	0.44	0.11	0.74	0.02	−0.62	0.03	0.17	0.05	0.99	0.13
Stomach	1	−0.10	0.03	−0.02	−2.43	−0.27	−1.63	−0.17	−0.84	−0.11	−0.17	−0.14	0.38	0.17	−1.21	−0.13	−0.49	−0.10	0.11	−0.18	0.48	0.23
	5	0.20	−0.11	0.55	0.89	−0.13	0.56	−0.13	0.22	−0.07	−0.01	0.08	0.20	−0.20	0.38	−0.11	0.09	−0.02	−0.06	0.16	0.36	−0.40
Melanoma	1	−0.01	−0.01	0.11	0.00	0.00	−0.01	−0.01	0.03	0.03	0.06	0.06	−0.09	−0.09	0.01	0.01	0.05	0.05	0.06	0.06	−0.13	−0.13
	5	0.00	0.00	0.15	0.02	0.02	0.03	0.03	0.04	0.04	0.03	0.03	−0.08	−0.08	0.03	0.03	0.04	0.04	0.01	0.01	−0.10	−0.10
Ovarian	1	−0.19	−0.13	0.04	0.36	0.06	0.03	0.00	−0.25	−0.13	−0.61	−0.28	−0.10	−0.16	−0.15	−0.05	−0.35	−0.21	−1.02	−0.32	0.47	−0.06
	5	0.18	0.06	0.35	0.28	0.01	−1.25	0.08	0.90	0.17	0.69	0.19	−0.16	−0.13	−0.82	0.12	2.15	0.21	−1.71	0.10	1.11	−0.26
Hodgkins	1	−0.10	−0.10	0.28	−0.02	−0.02	−0.04	−0.04	−0.10	−0.10	−0.19	−0.19	−0.34	−0.34	−0.07	−0.07	−0.14	−0.14	−0.26	−0.26	−0.39	−0.39
	5	−0.01	−0.01	0.01	−0.01	−0.01	−0.01	−0.01	−0.02	−0.02	−0.02	−0.02	0.00	0.00	−0.02	−0.02	−0.02	−0.02	−0.01	−0.01	0.00	0.00

a The five groups used for the age-group estimates were 18–44, 45–54, 55–64, 65–74 and 75+.

**Table 2B:** Differences between the estimates of survival of the reference model with the one with the minimum AIC and BIC respectively, for males as a whole population (standardised), males in age-groups or males aged 55, 65, 75 and 85 by type of cancer.

Cancer Type	Time (years)	Standardised			Group 1^a^		Group 2^a^		Group 3^a^		Group 4^a^		Group 5^a^		55		65		75		85	
		AIC	BIC	pp	AIC	BIC	AIC	BIC	AIC	BIC	AIC	BIC	AIC	BIC	AIC	BIC	AIC	BIC	AIC	BIC	AIC	BIC
Bladder	1	−0.14	0.02	0.29	−0.13	0.33	−0.13	0.30	−0.12	0.18	−0.12	0.00	−0.17	−0.05	−0.12	0.26	−0.12	0.11	−0.13	−0.11	−0.18	−0.04
	5	−0.02	−0.35	0.23	0.04	0.01	0.04	−0.01	0.03	−0.06	0.02	0.09	−0.06	−0.73	0.04	−0.04	0.02	−0.03	0.00	0.25	−0.09	−1.25
Lung	1	−0.25	−0.15	−0.11	−2.30	−0.13	−1.08	−0.10	0.74	−0.03	−0.82	−0.05	−0.10	−0.28	0.35	−0.06	−0.10	−0.02	0.07	−0.13	−0.45	−0.39
	5	0.08	0.06	0.25	−1.57	0.06	−0.49	0.03	0.80	−0.03	−0.58	−0.03	0.38	0.17	0.69	0.00	−0.07	−0.04	0.30	0.01	0.30	0.28
Colon	1	−0.09	−0.07	0.25	−0.74	0.24	0.44	0.07	0.27	−0.07	−0.45	−0.08	0.00	−0.10	0.83	−0.01	−0.71	−0.10	1.06	−0.04	−1.09	−0.14
	5	0.03	0.00	0.33	−2.69	−0.01	1.53	0.04	1.10	0.05	−1.09	−0.03	0.38	0.00	2.83	0.06	−1.81	0.03	2.88	−0.10	−1.74	0.08
Rectum	1	−0.05	−0.04	0.09	−0.28	1.13	0.09	0.50	0.17	−0.09	−0.44	−0.20	0.18	−0.06	0.25	0.17	−0.08	−0.24	−0.32	−0.10	0.42	−0.05
	5	−0.02	−0.10	0.25	−2.70	−0.32	1.01	−0.19	0.44	−0.06	−0.51	0.00	0.11	−0.18	2.17	−0.12	−1.94	−0.01	1.46	−0.01	−0.36	−0.32
Stomach	1	0.05	0.13	0.62	−1.07	−0.03	−0.81	−0.01	−0.41	0.01	0.08	−0.03	0.35	0.29	−0.63	0.01	−0.19	0.00	0.34	−0.04	0.31	0.52
	5	0.07	−0.08	0.29	0.19	0.09	0.18	0.02	0.14	−0.01	0.05	0.08	0.05	−0.21	0.17	−0.01	0.11	0.01	−0.01	0.12	0.11	−0.49
Melanoma	1	−0.01	−0.05	0.17	−0.01	−0.07	−0.17	−0.03	0.31	0.05	−0.02	0.11	−0.17	−0.27	0.12	0.00	0.23	0.09	−0.22	0.08	−0.17	−0.44
	5	−0.13	−0.15	0.00	−0.02	−0.03	−0.26	−0.05	0.41	−0.02	−0.21	−0.02	−0.48	−0.50	0.19	−0.04	0.12	0.00	−0.13	−0.09	−0.71	−0.74
Prostate	1	−0.20	−0.19	−0.10	−0.10	−0.07	−0.04	−0.04	−0.09	−0.06	−0.21	−0.24	−1.17	−1.04	−0.07	−0.06	−0.04	−0.04	−0.16	−0.11	−0.55	−0.65
	5	−0.01	0.01	0.08	−0.36	−0.02	0.24	0.00	−0.40	0.00	0.46	0.00	−0.28	0.12	−0.01	−0.01	0.08	0.00	−0.41	−0.01	0.98	0.06
Hodgkins	1	0.13	0.13	0.27	0.01	0.01	0.06	0.06	0.16	0.16	0.32	0.32	0.52	0.52	0.10	0.10	0.24	0.24	0.44	0.44	0.59	0.59
	5	0.00	0.00	0.24	0.00	0.00	0.00	0.00	0.01	0.01	0.01	0.01	−0.01	−0.01	0.01	0.01	0.01	0.01	0.00	0.00	−0.02	−0.02

a The five groups used for the age-group estimates were 18–44, 45–54, 55–64, 65–74 and 75+. Age-group estimates for prostate cancer were however obtained for the groups 18–54, 55–64, 65–74, 75–84 and 85+.

Differences for the age-group estimates are slightly larger (Table 2A, Table 2B), however most of them are lower or close to 1 percentage point. For females, the median and mean value for absolute differences with the AIC model are equal to 0.083 and 0.229 percentage points respectively. Differences for the younger groups are higher but this can be partly explained by the smaller number of patients in these groups.

**Fig. 2:**
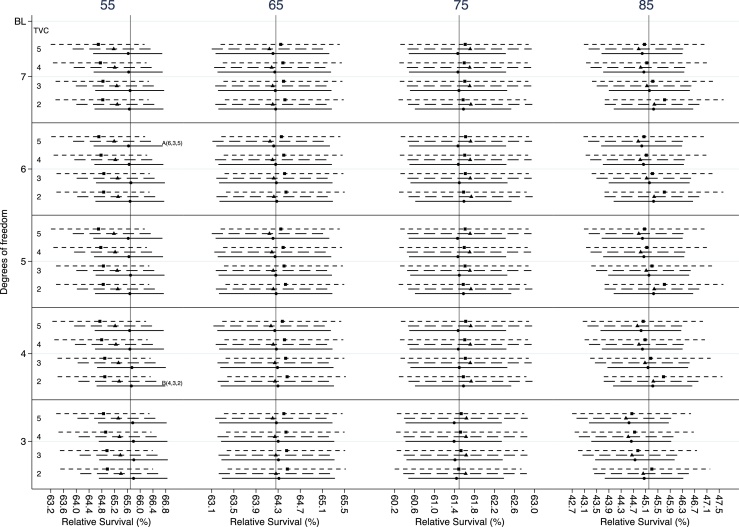
Estimates for 5-year relative survival for female patients with colon cancer diagnosed at 55, 65, 75 and 85 years. The dots represent the point estimates and the lines either side the 95% confidence intervals. The vertical line, in each plot, represent the estimate obtained by the reference model. Solid, dash and dot- ted horizontal lines represent 3, 4 and 5 degrees of freedom, respectively, for the main effect of age. (BL: degrees of freedom for the baseline excess hazard, TVC: degrees of freedom for the time-dependent effect of age, A: model chosen by AIC, B: model chosen by BIC).

Age-specific estimates are, due to their nature, more sensitive to the number of knots, and more caution is needed when we choose df for the splines. However, the median and mean absolute differences between the reference model and the selection criteria remain very low. Differences with the AIC model for females have a median of 0.108 and a mean of 0.271 percentage points. Similarly, the median and mean absolute difference with the BIC model are equal to 0.106 and 0.140 percentage points respectively. Age-specific estimates for females with colon cancer are given in Fig. 2. The 5-year relative survival of a patient diagnosed at the age of 55 and 85 years old vary the most under different scenarios in comparison with the other ages. A similar pattern was observed for the 1-year relative survival estimates (Appendix B, Fig B1). More than 3 df might be needed to model the baseline excess hazard (Fig. 2). However, the estimates are not over-sensitive to the number of df and differences in relative survival estimates between the reference model and the models chosen by AIC (i.e. model with 6,3,5 df for the baseline excess hazard, the main and the time-dependent effect of age respectively) and BIC (i.e. model with 4,3,2 df for the baseline excess hazard, the main and the time-dependent effect of age respectively) criteria are small (Table 2A).

**Fig. 3:**
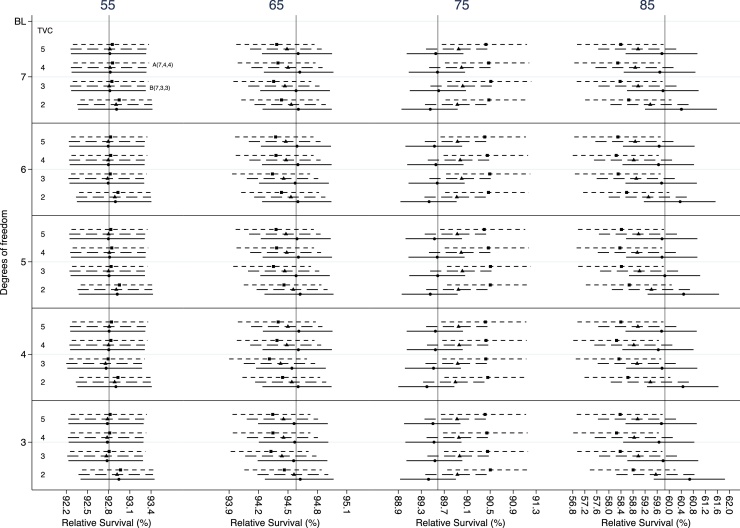
Estimates for 5-year relative survival for male patients with prostate diagnosed at 55, 65, 75 and 85 years. The dots represent the point estimates and the lines either side the 95% confidence intervals. The vertical line, in each plot, represent the estimate obtained by the reference model. Solid, dash and dotted horizontal lines represent 3, 4 and 5 degrees of freedom, respectively, for the main effect of age. (BL: de- grees of freedom for the baseline excess hazard, TVC: degrees of freedom for the time-dependent effect of age, A: model chosen by AIC, B: model chosen by BIC).

A bigger variation is observed in 5-year age-specific estimates for prostate cancer (Fig. 3). The largest differences are seen among different df for the main effect of age and for 75 and 85 years old estimates. The selection criteria suggest that a more complicated model will be more appropriate. The AIC chooses the model with 7,4 and 4 df for the baseline excess hazard, the main and the time-dependent effect of age respectively. The equivalent df for the BIC are 7,3 and 3 respectively. However, the differences between the reference model and the models chosen by the selection criteria remains lower than 1 percentage point for all ages (Table 2B). Smaller differences were observed for 1-year relative survival (Appendix B, Fig B2).

**Fig. 4:**
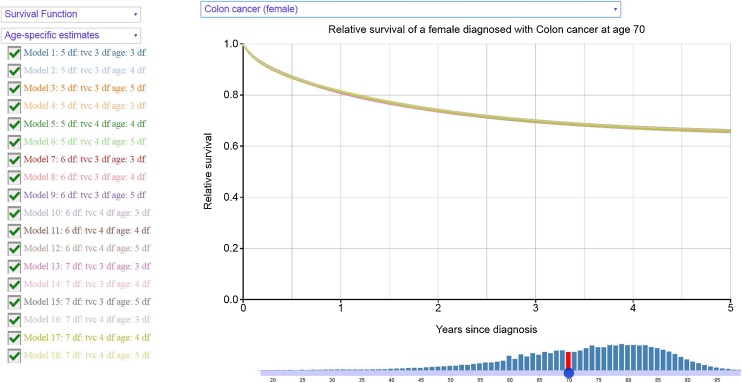
Snapshot from the web-based interactive graphs.

The results are also presented in web-based interactive graphs available at http://pclambert.net/interactivegraphs/model_sensitivity/model_sensitivity, a snapshot of which can be found in Fig. 4. Users can compare the estimates for a range of models by clicking a box. Both estimates of relative survival and excess hazard functions over years since diagnosis are given and there is also an option to choose marginal or age-specific estimates. For example, by moving the slider in the age histogram that can been seen in the bottom of Fig. 4, estimates of relative survival for a 70 years old female with colon cancer are presented.

## 4 Discussion

We performed sensitivity analyses to assess the sensitivity of estimates obtained from FPMs on the number of knots chosen for the splines by using relative survival as an example. Even though we used relative survival to conduct the sensitivity analysis, our conclusions can also be applied to other settings such as FPMs that do not incorporate the expected mortality. We have also developed web- based interactive graphs that allow the comparison of estimates from different models. We advocate the use of interactive graphs for reporting findings as they allow for additional exploration and improve understanding of results [[29], [30], [31]].

The results of the sensitivity analysis indicate differences between the reference model and the models chosen by the selection criteria for the age-standardised estimates are negligible. Age-group relative survival is less stable, among models, with slightly larger differences for the youngest group, of whom there are fewer patients. For most of the cancers the df for the main and time-dependent effect of age vary the least whereas 4 df seem adequate to capture the underlying baseline excess hazard.

Age-specific estimates are more sensitive to the number of knots chosen but perform well. For most of the cancer types, higher differentiation is observed among models with different df for the splines of the main effect of age. However, the differences between the reference model and the models chosen by the selection criteria remain low. Slightly larger differences were observed for the age of 55 years old at diagnosis for male patients with colon and rectum cancers but this can partly be explained by the smaller number of patients at that age. For such cancer types choosing 3 df seems insufficient for capturing the shape of the main effect of age and more are required. More caution is needed when interest is in age-specific estimates and the choice for the df for the splines should be given further thought, with the hazard and survival function of the cancer considered.

In general, when too few df are specified, the estimation may become problematic and is better to specify more knots. In the models with the highest df used for the splines, the estimates are very close with the estimates of the reference model and the models selected by the AIC and BIC criteria. Of course too many df should be avoided as they can result in overfitting, especially for datasets with a small number of observations.

Our results are consistent with results from simulation studies that pointed out that estimates are not influenced noticeably by the number of knots, as long as a sensible number of knots is selected [13,14]. In large datasets, the AIC and BIC will select models with high df when a lower value for the df provide a similar fit. In our analysis, the selection criteria chose more complicated models but the differences in the estimates between the models selected by the selection criteria and the reference model were negligible.

A major strength of our analysis is the large number of patients involved in each cancer type that enable reliable conclusions. The cancer types chosen, allow the assessment of FPMs for cancers with varying prognosis and other characteristics. Furthermore, the wide range of df selected for the splines and the 60 FPMs for each type of cancer provided a thorough evaluation of obtained estimates.

Although this study has noteworthy strengths, we should acknowledge potential limitations. The population in our data includes only patients enrolled in one of the cancer registries in England. Populations from other countries may have different characteristics that affect their survival. Moreover, patients above the 98th percentile and below the 2nd percentile of the age distribution were forced to take the same relative survival as those patients at these respective cut-off points. Using these constraints leads to more stable estimates in the extremes where there is less data and helps with some model convergence issues.

## 5 Conclusions

FPMs overcome some of the limitations that traditional methods encounter and they have the ability to capture the shape of complex hazard functions by using restricted cubic splines. Time-dependent effects can easily be incorporated in FPMs. We showed that age-specific, age-group and age-standardised estimates are not over-sensitive to the specified number of knots and that the use of restricted cubic splines is a valid approach for time-to-event data. We also highly recommend the use of the webtool as an easy way to visualise the differences across different scenarios.

## Declarations of interest

None.

## Funding

This work was supported by Cancer Research UK [Grant number C1483/A18262].

## Authorship contribution

All authors made substantial contributions to conception and design, acquisition of data, or analysis and interpretation of data and participated in drafting the article. All authors interpreted the data, critically revised the manuscript and gave final approval of the version to be published.
