# Supplementary material for: Robustness of individual and marginal model-based estimates: A sensitivity analysis of flexible parametric models
Source: Cancer Epidemiol. 2019 Feb;58:17–24. doi: 10.1016/j.canep.2018.10.017 (PMC6363964; doi:10.1016/j.canep.2018.10.017)
Supplement: Supplementary file 1 [file mmc1.pdf]

Appendix A

Splines are flexible mathematical functions defined by piecewise polynomials. The most common used splines are the cubic spines that use cubic polynomials between the knots and under constraints produce a smooth function. The points at which the polynomials join are called knots.The first and the last of the knots are known as boundary knots.

An extension of cubic splines are restricted cubic splines which are forced to be linear before the first knot and after the final knot. Fitted as a linear function of $K-1$ derived covariates, where K is the number of knots restricted cubic splines are given by

$$s(\ln(t)|\gamma, k_0) = \gamma_0 + \gamma_1 z_1 + ... + \gamma_{K-1} z_{K-1}.$$

The derived variables z_i are calculated as

$$z_1 = x$$

$$z_i = (x - k_j^3)_+ - \lambda_j (x - k_{min})_+^3 - (1 - \lambda_j)(x - k_{max})_+^3,$$

where $u_+ = u$ if $u \leq 0$, k_{min} and k_{max} are the position of the first and the last knot and $\lambda_j = \dfrac{k_{max} - k_j}{k_{max} - k_{min}}$.
