# Supplementary material for: Robustness of individual and marginal model-based estimates: A sensitivity analysis of flexible parametric models
Source: Cancer Epidemiol. 2019 Feb;58:17–24. doi: 10.1016/j.canep.2018.10.017 (PMC6363964; doi:10.1016/j.canep.2018.10.017)
Supplement: Supplementary file 2 [file mmc2.pdf]

Appendix B

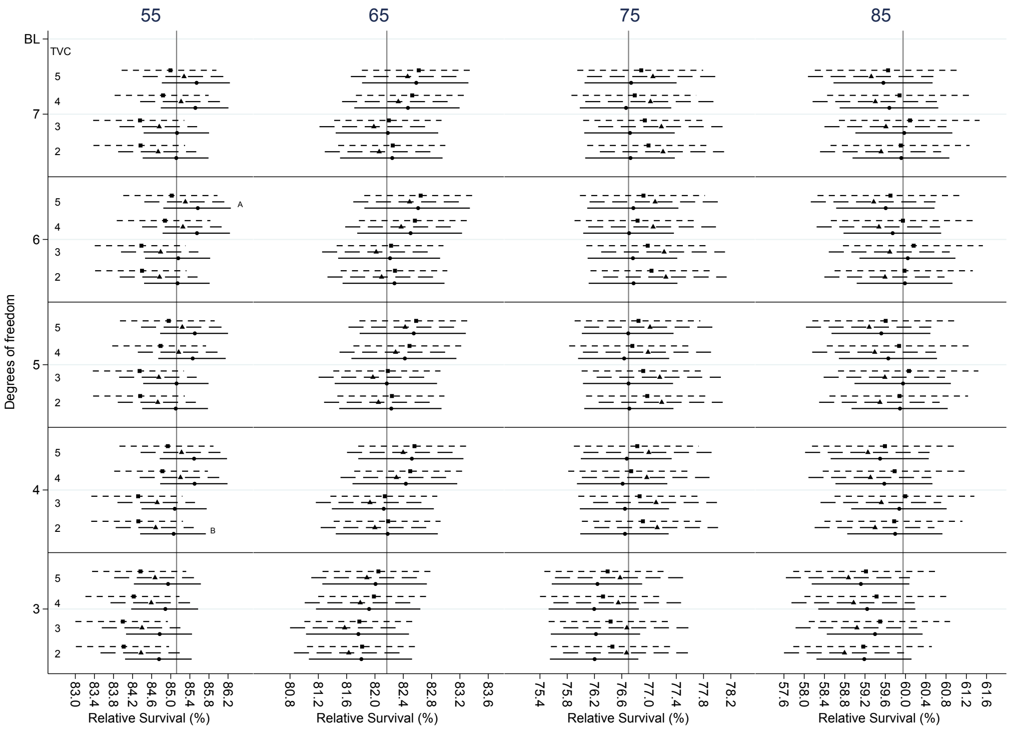

Fig. B1: Estimates for 1-year relative survival for female patients with colon cancer diagnosed at 55, 65, 75 and 85 years. The dots represent the point estimates and the lines either side the 95% confidence intervals. The vertical line, in each plot, represent the estimate obtained by the reference model. Solid, dash and dotted horizontal lines represent 3, 4 and 5 degrees of freedom, respectively, for the main effect of age. (BL: degrees of freedom for the baseline excess hazard, TVC: degrees of freedom for the time-dependent effect of age, A: model chosen by AIC, B: model chosen by BIC)

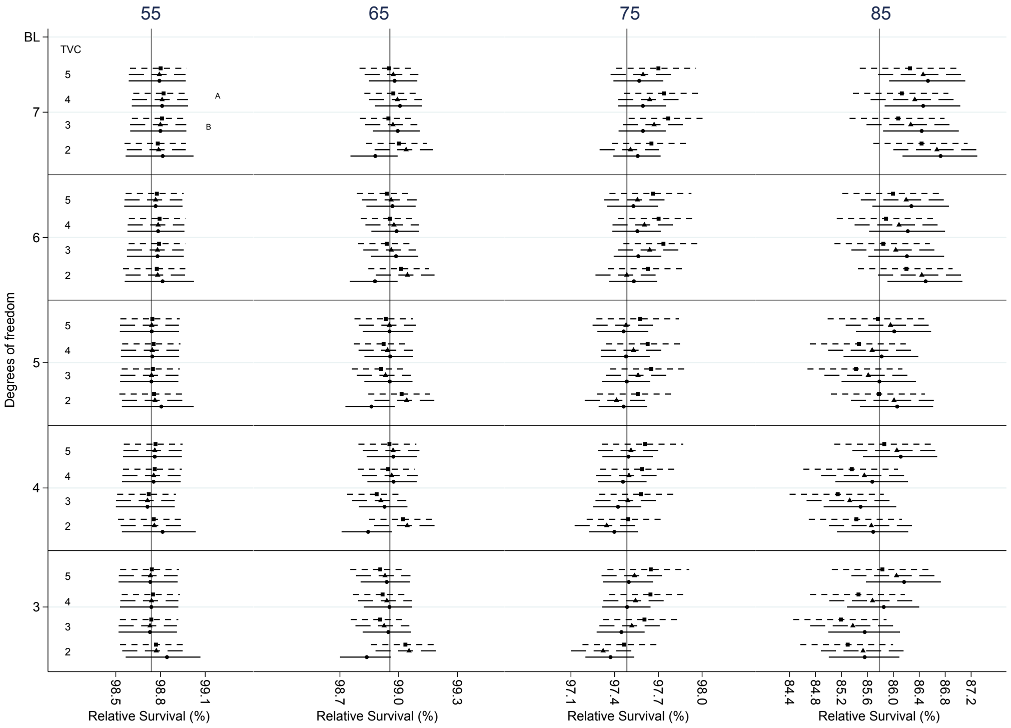

Fig. B2: Estimates for 1-year relative survival for male patients with prostate diagnosed at 55, 65, 75 and 85 years. The dots represent the point estimates and the lines either side the 95% confidence intervals. The vertical line, in each plot, represent the estimate obtained by the reference model. Solid, dash and dotted horizontal lines represent 3, 4 and 5 degrees of freedom, respectively, for the main effect of age. (BL: degrees of freedom for the baseline excess hazard, TVC: degrees of freedom for the time-dependent effect of age, A: model chosen by AIC, B: model chosen by BIC)
