# Supplementary material for: Robustness of individual and marginal model-based estimates: A sensitivity analysis of flexible parametric models
Source: Cancer Epidemiol. 2019 Feb;58:17–24. doi: 10.1016/j.canep.2018.10.017 (PMC6363964; doi:10.1016/j.canep.2018.10.017)
Supplement: Supplementary file 3 [file mmc3.pdf]

Appendix C

Below we give an example on the Stata code that was used to obtain non-parametric and model-based estimates of relative survival.

First, we need to declare our data as survival data. This is done in Stata with the `stset` command. To conduct a period analysis, additional information should be given on the beginning and the end of the period window. These can be specified by options `enter` and `exit`. In the following example, patients enter the follow-up at diagnosis (`dx`) and they remain until they die or until the censoring time (`dexit`). However, the analysis includes only the follow-up time during the period window, from the beginning of 2011 to the end of 2013.

```
stset dexit,  failure(status==1) enter(time mdy(1,1,2011)) exit(time mdy(12,31,2013)) ///
origin(dx) id(patid) scale(365.24)
```

1 Non-parametric estimates

Non-parametric estimates can be estimated using the `strs` command. The `popmort.dta` file includes information on the expected mortality stratified by sex, year and age. Variable `standweight` contains weights that in our application were calculated based on the number of patients in each age-group. More information on the syntax of `strs` command can be found in the paper by Dickman and Coviello (2015).

```
strs using popmort [iw=standweight] if sex == ‘sex’, br(0(‘=1/12’)5) standstrata(agegrp) ///
pohar mergeby(sex  year  age) diagyear(yeardiag) attage(age) attyear(year) ///
diagage(agediag) survprob(survprob) savstand(agestandIn,replace) notables
```

2 Model-based estimates

First, we need to merge to the dataset the life table with information on the expected mortality of the general population stratified by specific variables. In our study, these were sex, age and calendar year.

To deal with less stable estimates in the extremes we forced patients below the 2nd percentile of the age distribution to have the same relative survival as patients of this cut-off. This can be done with the following commands:

```
//Calculate percentiles corresponding to the 2nd percentile of the age at diagnosis
//distribution (variable agediag)
_pctile agediag, per(2)
//Store it in local variable with name age_cutoff_low
local age_cutoff_low ‘r(r1)’
//Generate a new variable(ageadj) that is equal to age at diagnosis if age at diagnosis
//is higher than the age that corresponds to this cut-off.
//If not, then replace it with the value of the cut-off.
gen ageadj = cond(agediag<‘age_cutoff_low’,‘age_cutoff_low’,agediag)
//Do the same for the upper bound of the 98th percentile
_pctile agediag, per(98)
local age_cutoff_high ‘r(r1)’
replace ageadj = cond(ageadj>‘age_cutoff_high’,‘age_cutoff_high’,ageadj)
```

To estimate the restricted cubic splines for the main effect of age use the command `rcsgen`. Option `df()` specifies the df for the splines. In the reference model this was equal to 3. New variables `agercs1, agercs2` and `agercs3` will be generated for the splines. The `orthog` option is used to orthogonalize the generated

spline variables using Gram-Schmidt orthogonalization and therefore enhance stability when fitting the model.

```
rcsgen ageadj, df(3) gen(agercs) orthog
```

The reference FPM can then be fitted as:

```
stpm2 agercs1 agercs2 agercs3, scale(hazard) df(5) bhazard(rate) ///
tvc(agercs1 agercs2 agercs3) dftvc(3)
```

In this model, 5 df were used to model the baseline hazard function (specified by option `df()`). The time-dependent effects of age were also included in the model (option `tvc()`) with 3 df (option `dftvc()`). The option `bhazard(rate)` invokes that a relative survival model will be fitted and variable rate has the information on the expected mortality rate.

Predictions of age-standardised relative survival can then easily be obtained.

```
predict surv_stand, meansurv timevar(timevar)
```

A new variable `surv_stand` will be generated. Option `meansurv` specifies that a population averaged survival function is requested. With `meansurv`, first we obtain predictions for each individual in our study population and the we calculate an average of those. `timevar` specifies the time variable used for predictions.

Predictions for age-group standardised relative survival can be obtained just by including the `if` option (variable `agegrp` denotes the 5 age groups):

```
foreach group in 1 2 3 4 5 {
predict surv_stand_AgeGroup`group' if agegrp==`group' , meansurv timevar(timevar)
 }
```

Predictions for age-specific relative survival can also be derived by a similar way. For example for age 65:

```
predict surv_stand_Age65, survival at(rcsage1 `=c1' rcsage2 `=c2' rcsage3 `=c3' ) ///
timevar(timevar)
```

where `` `=c1' `` , `` `=c2' `` and `` `=c3' `` yields the values of the spline variables for the age of 65 so that we make sure we use the same knot locations and projection matrix.
